# Mutation and Epistasis in Influenza Virus Evolution

**DOI:** 10.3390/v10080407

**Published:** 2018-08-03

**Authors:** Daniel M. Lyons, Adam S. Lauring

**Affiliations:** 1 Department of Ecology and Evolutionary Biology, University of Michigan, Ann Arbor, MI 48109, USA; lyonsdm@med.umich.edu; 2 Division of Infectious Diseases, Department of Internal Medicine, University of Michigan, Ann Arbor, MI 48109, USA; 3 Department of Microbiology and Immunology, University of Michigan, Ann Arbor, MI 48109, USA

**Keywords:** influenza, mutation, epistasis, evolution, reassortment

## Abstract

Influenza remains a persistent public health challenge, because the rapid evolution of influenza viruses has led to marginal vaccine efficacy, antiviral resistance, and the annual emergence of novel strains. This evolvability is driven, in part, by the virus’s capacity to generate diversity through mutation and reassortment. Because many new traits require multiple mutations and mutations are frequently combined by reassortment, epistatic interactions between mutations play an important role in influenza virus evolution. While mutation and epistasis are fundamental to the adaptability of influenza viruses, they also constrain the evolutionary process in important ways. Here, we review recent work on mutational effects and epistasis in influenza viruses.

## 1. Introduction

Influenza viruses infect a large number of hosts, have high mutation rates, and frequently reassort. As a result, they have a tremendous capacity to explore a large number of potential sequences. Indeed, the ability of influenza populations to adapt to new hosts and to escape the immune system seems unlimited. However, mutations are often deleterious, which presents a barrier to viral adaptation. Furthermore, interactions between mutations, or epistasis, determine the mutational paths available and can make some adaptations inaccessible. Understanding how mutation and epistasis present both barriers and opportunities for influenza virus evolution is essential in predicting viral evolution and designing better vaccines and antivirals.

## 2. Effects of Single Mutations

The distribution of mutational fitness effects (DMFE) reveals the extent of genetic constraint on the influenza virus genome, how constraints vary between and within influenza proteins, and the structural and functional impacts of mutations. Mutational fitness effects can be measured in a variety of ways. Site-directed mutagenesis allows for precise control of the identity and number of mutations created. This is usually combined with a competitive fitness assay that provides a precise measure of the fitness of a given mutant relative to the wild-type [1,2], an approach that is reliable, but labor-intensive. Deep mutational scanning (DMS) combines large-scale mutagenesis with bulk fitness measurements of a mutagenized library through next-generation sequencing [3]. This method allows for a nearly complete sampling of single mutations across a gene, but typically does not precisely control the number of mutations in a clone. Therefore, the fitness of a mutation in DMS represents its average effect across several unknown backgrounds (but see Reference [4]). Furthermore, DMS is less sensitive for lethal and low-fitness mutations. A third strategy is to monitor viral populations in natural infections by deep sequencing. This interrogates the effect of mutations in a realistic environment [5,6,7,8], but cannot control for the effect of genetic background or assign fitness effects to individual mutations. These three approaches therefore provide complementary insights into the DMFE.

### 2.1. Genome-Wide Distribution of Mutational Fitness Effects

Site-directed mutagenesis has been used to characterize the genome-wide DMFE of single mutations in a variety of viruses [1]. Our laboratory applied this technique to an H1N1 influenza strain [2]. We generated a library of 95 randomly selected point mutations distributed across the influenza genome. We also generated 33 additional mutations in the segments encoding the hemagglutinin (HA) and neuraminidase (NA) proteins to compare the DMFE of these surface-exposed antigenic proteins (*n* = 57) to the internal proteins encoded by the remaining six segments (*n* = 71).

We measured fitness relative to wild-type in a pairwise competition assay and used repeated transfection to distinguish true lethal mutations (fitness = 0) from failed viral rescue. In our dataset, 31.6% of all mutations were lethal. Approximately 40% of all viable mutations were highly detrimental (<0.85), 50% mildly detrimental or neutral (0.85–1.05), and only seven were beneficial (>1.05). The fitness among all viable mutations ranged from 0.26–1.13 with a mean of 0.82. Nonsynonymous mutations were more deleterious than synonymous mutations, consistent with reduced genetic constraint at the level of RNA relative to protein. Two of the three noncoding mutations were lethal, consistent with the conserved roles of these regions in RNA packaging and genome replication [9,10].

In general, the DMFE for influenza viruses is similar to those documented for other viruses with a variety of genomic structures, from single-stranded RNA viruses to DNA phages [1,2,11,12,13,14]. The lethal fraction for influenza viruses falls within the 20–40% range observed for other viruses. When scaled to exponential growth rate—the fitness surrogate in many studies—the average fitness effect in influenza viruses is 12%, squarely within the 10–13% range found in other viruses [1,2]. These large effects stand in marked contrast to those seen in cellular organisms [15] and may reflect shared genetic constraints across viruses, possibly related to the small size of their genomes [16]. 

While genome-wide patterns in the DMFE are similar across viruses, the DMFE varies between and within individual influenza genes. The antigenic proteins, HA and NA, evolve much more rapidly than other influenza proteins. This rapid evolution could be due to a history of stronger positive selection and/or an inherently greater mutational tolerance [17]. In our comparative study, we found that the antigenic proteins were generally more tolerant of mutation. The mean fitness of mutations in the surface proteins (0.88) was higher than for the internal proteins (0.78). Furthermore, the head region of HA, which is immunodominant and exhibits the greatest sequence diversity, had a higher mean fitness (0.77) than the stalk region (0.56), which exhibits lower sequence diversity. Other groups have also documented the relative mutational tolerance of HA. For example, Heaton et al. mutagenized the entire influenza genome with 15-nucleotide insertions [18]. A disproportionate number of recovered variants had insertions in the head region of HA (7/20 recovered variants). DMS studies have further confirmed the mutational tolerance of rapidly evolving HA regions, revealing high tolerance in antigenic domains and very low tolerance in the slower-evolving HA receptor-binding pocket and stalk domain [19,20]. These data suggest that mutational tolerance in HA and NA, particularly in the HA head, contributes to their greater evolutionary potential. It will be interesting to compare these patterns to those in the major antigenic proteins of other viruses [21]. 

In influenza viruses, other protein regions with immunodominant epitopes do not always recapitulate the trends in HA. For example, the solvent-exposed region of the nonstructural protein 1 (NS1), which likely interacts with host proteins to modulate immune responses, also has greater tolerance to insertions [18]. However, immune-targeted sites in the nucleoprotein (NP) do not show unusually high mutational tolerance [20,22]. Perhaps these NP regions are less tolerant because they experience lower diversifying selection from the immune system. Alternatively, these sites could be inherently more constrained. Understanding the causes and consequences of varied mutational tolerance is highly relevant to vaccine design, as the lower mutational tolerance of the HA receptor-binding pocket and stalk make them attractive targets for a universal vaccine [23].

Overall, the vast majority of mutations in influenza viruses are lethal or deleterious. Given the virus’s high mutation rate of two–three per genome replicated, a large proportion of newly replicated genomes will contain a lethal mutation, and many more will harbor one or more deleterious mutations [2,24]. Within hosts, the constraints of deleterious mutations are manifest as high levels of purifying selection and limited genetic variation [5,25,26,27,28,29,30,31]. Deleterious mutations also impact influenza evolution at the global scale, because purifying selection does not always efficiently purge them from the population. Deleterious mutations may reach fixation by drift (e.g., during transmission bottlenecks) or by hitchhiking with adaptive mutations. Influenza virus phylogenies show a high deleterious mutation load [32], and models suggest that this load can slow antigenic evolution [33,34].

### 2.2. Deep Mutational Scanning of Influenza Proteins

While site-directed mutagenesis has provided an overview of the genome-wide DMFE, DMS can interrogate nearly all amino acid substitutions in a single protein. In DMS studies, fitness is usually calculated as the change in frequency of a mutation in a pool of variants before and after passage or selection, relative to wild-type. This method is analogous to pairwise competition assays, and fitness measurements across studies are well correlated [2,35]. DMS studies by Bloom and colleagues include saturation mutagenesis of the HA and NP proteins from H1N1 and H3N2 strains [19,20,22,36,37]. Studies by Sun and colleagues investigated many substitutions in nearly all sites in the six other influenza proteins [4,38,39,40,41,42,43]. We are now close to a complete map of the fitness effects of all possible amino acid substitutions for an entire influenza virus genome.

Both sets of DMS studies clearly show that mutational tolerance varies widely across sites within a protein; some sites strongly prefer a single amino acid and others accept many different amino acids. The effect of any particular amino acid substitution is also highly site-specific. Wu et al. investigated the link between protein stability and mutational effects in PA to shed light on why constraints may vary across sites [4]. They found two categories of amino acid residues: those in which substitutions affected overall protein stability and those in which substitutions were detrimental but did not affect stability. The latter were termed “functional” residues as they likely affected enzymatic functions of a protein (e.g., polymerase activity) or important protein–protein interactions (e.g., solvent-exposed sites).

Broad mutational categories such as transversions and transitions can also capture functional constraints on influenza proteins [35]. Using available DMS data in HA and NP [22,36,37], we found that amino acid substitutions that are only accessible by transition mutations are more detrimental than those accessible by transversions. This suggests that selection against transversion mutations is a significant contributor to the observed transition–transversion substitution bias in viruses [44]. Radical changes in biochemical properties such as size, polarity, and charge, also have more detrimental fitness effects in HA and NP. Interestingly, although transversions are more likely to cause such radical changes, this does not completely explain their more detrimental effects. Thus, we have much to learn about the biological basis for mutational fitness effects. 

More recent studies have used DMS in innovative ways. DMS-informed, site-specific, and parameter-free evolutionary models dramatically improve the fit of phylogenies [22,37,45] and the inference of sites under positive selection [46,47]. Another promising avenue is the application of DMS to phenotypes other than fitness [40,41]. Du et al. used DMS to identify mutations that increase IFN sensitivity while preserving replicative fitness and immunogenicity, leading to a potentially safe and effective vaccine strain [39]. Bloom and colleagues have used DMS to study the potential mutational pathways of antibody escape and identified regions of HA with low escape potential [48,49]. 

## 3. Epistasis

**Figure 1:**
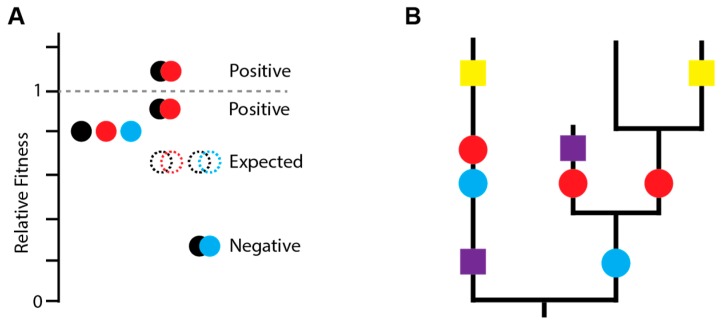
Identifying epistasis. (**A**) Epistasis is defined based on deviation from the expected fitness assuming independent multiplicative effects. Black, red, and blue mutations are each deleterious (filled individual circles) and the expected fitness of pairwise combinations (empty circles) is shown. Black and red mutations exhibit positive epistasis because combining them is not as deleterious as expected and may even increase fitness. Black and blue mutations exhibit negative epistasis because combining them is more deleterious than expected. (**B**) Positive epistasis can be inferred phylogenetically. Red and blue mutations interact positively as they are always present together and occur closely in time. In contrast, yellow and purple mutations do not occur closely in time and are not necessarily present together.

Mutations do not occur on a universal genetic background and may arise together in the same genome. The influenza virus also exhibits reassortment, a form of viral sex that combines mutations on different segments from different genetic backgrounds. Epistasis (ϵ) refers to the genetic interactions between two or more mutations in a genome. It underlies the genetic basis of complex traits and shapes many evolutionary processes, from speciation to the adaptability of populations [50]. However, much of the work on epistasis in viruses is relatively recent [51]. Epistasis is most commonly defined as the difference between the observed fitness of the genome with both mutations i and j (w_ij_) and the expected fitness given independent multiplicative effects of each single mutation (Figure 1A) [52]. Thus, ϵ = w_ij_ − w_i_ × w_j_, where ϵ = 0 indicates no genetic interaction. Negative epistasis (ϵ < 0) occurs when the fitness of a double mutant is less than expected. Positive epistasis (ϵ > 0) occurs when the fitness of the double mutant is greater than expected. As we mainly discuss epistasis as either positive or negative below, we refer the reader to Reference [52] for a more detailed review of epistasis terminology.

### 3.1. Detecting and Measuring Epistasis

Experimental assays for measuring the fitness effects of single mutations can also be used to determine the sign (positive or negative) and magnitude of epistasis between two or more mutations. Previous studies have employed site-directed mutagenesis to study interactions among small numbers of mutations, usually those implicated in adaptation to immune pressure or antiviral drugs [53,54,55]. As above, the advantage of site-directed mutagenesis is that one can precisely quantify the epistatic interactions between chosen mutations. Mutations can also be introduced anywhere in the genome, allowing one to study both between- and within-gene epistasis. 

In contrast, DMS offers the throughput necessary to study epistasis more broadly [56,57]. It is most useful for studies of within-gene epistasis, particularly for small contiguous regions that can be sequenced in a single read. Alternatively, DMS studies can be compared across different genetic backgrounds [19,22]. Shifts in mutational effects at a given site across different genetic backgrounds reflect epistatic interactions involving that site. However, comparative DMS studies can only detect epistatic interactions involving at least one divergent site and cannot precisely identify the interacting mutations. Another comparative approach is to experimentally “replay” evolution in different genetic backgrounds to examine the influence of epistasis on evolutionary trajectories [58].

Phylogenetic inference allows one to identify epistatic interactions in the virus’s natural replication environment. One approach is to identify coevolving sites [59,60]. If substitutions at one site are followed by second site substitutions more quickly than expected by chance, these substitutions likely enhance each other’s beneficial effects (Figure 1B) [61]. This approach can only detect positive epistasis and has limited power for rarer polymorphisms and weaker epistatic interactions. Furthermore, it has typically been applied to studies of within-gene epistasis, given the added complexity of reassortment and the computational costs of genome-wide scans (but see Reference [62]). Phylogenetic inference of between-gene epistasis in influenza relies on observed patterns of reassortment. Here, nonrandom patterns of reassortment among genome segments suggest incompatible interactions [63]. These incompatibilities can also be detected as accelerated rates of evolution in reassortant lineages, as the newly combined segments adapt to their new genetic environment [64]. While these studies identify gene-level epistasis, they typically do not identify the interacting sites.

### 3.2. General Epistatic Patterns in Influenza Viruses

**Figure 2:**
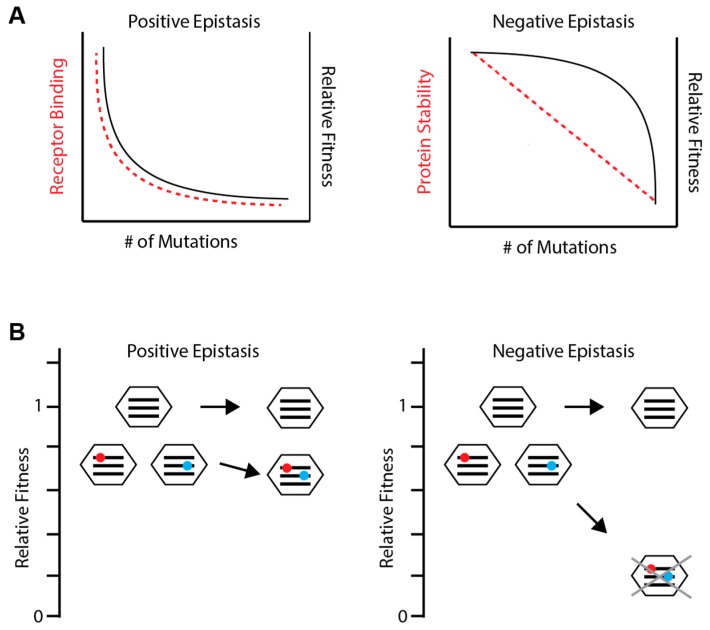
Patterns of epistasis. (**A**) Distinct epistatic patterns are predicted based on how phenotypic effects (red dashed lines) correspond to fitness effects (black solid lines). Small genomes may encode a single mechanism for a particular function, such as receptor binding in hemagglutinin (HA) (left). Without a backup mechanism, initial mutations have a large impact on receptor binding and fitness. Additional mutations have little further impact, as the function has already been destroyed, resulting in positive epistasis. Other phenotypes, like protein stability, can be reduced without affecting fitness until a threshold is reached (right). Thus, each additional mutation impacts protein stability similarly but increasingly impacts fitness, resulting in negative epistasis. (**B**) A viral population consisting of an unmutated genotype and two variants each with a slightly deleterious mutation (red and blue circles) on different segments (black lines). Reassortment between the two variants can combine the two deleterious mutations. If epistasis is positive (left), the reassortant will have higher fitness than expected and the deleterious mutations may persist in the population, lowering the average fitness of the population. If epistasis is negative (right), the reassortant will be quickly purged from the population, leaving the unmutated genotype and raising the average fitness of the population.

Recent studies have elucidated patterns of within-gene epistasis. Comparative DMS of NP and HA in H3N2 and H1N1 backgrounds have found that both short-range physical interactions and long-range functional interactions within these proteins are common [19,37]. Phylogenetic studies also find many long-range epistatic interactions [59,65]. Additionally, sites exhibiting epistasis cluster with each other, which can be explained by structural changes affecting a particular region of the protein [19,37]. Less is known about the type and magnitude of epistasis. A DMS study of 11 sites in the receptor-binding region of HA found positive epistasis to be ubiquitous [56]. However, studies in other taxa show that a protein stability threshold generally leads to negative epistasis across entire proteins (Figure 2A) [66,67,68,69,70].

Due to the limitations outlined above, there is little empirical work on the general patterns of epistasis between genes or genome-wide in any organism. Thus, our understanding of the influenza virus is mostly based on theoretical predictions (Figure 2A). One theory holds that epistasis depends on genome complexity and the extent of functional redundancy, which is limited in viruses with small genomes [16,71]. Here, the first mutation may have a large effect, but the impact of additional mutations is smaller, since they cannot further break functions already broken by the first mutation. This is positive epistasis. In contrast, viruses or organisms with larger genomes may have redundant pathways, which tend to buffer the impact of single mutations but less so for multiple mutations, leading to negative epistasis. In contrast, other models suggest that high mutation rates can select for distinct mechanisms that buffer the impact of single mutations and lead to negative epistasis, even in simple genomes [66,72,73,74]. For example, if fitness is reduced only when an underlying phenotype reaches a threshold, then the full deleterious impact of mutations affecting that phenotype will only be revealed when enough mutations accumulate to cross the threshold, resulting in negative epistasis [66].

The theoretical costs and benefits of reassortment largely depend on the type and magnitude of epistasis between mutations on different segments. Reassortment is advantageous in the setting of negative epistasis because combining deleterious mutations through reassortment will accelerate the rate at which they are purged from a population (Figure 2B) [75,76,77]. Conversely, positive epistasis slows the rate at which deleterious mutations are purged, making reassortment disadvantageous. Reassortment also underlies the process of antigenic shift and the associated spread of avian and swine viruses to humans [78,79,80]. However, segments do not reassort freely [63,81], and differential pairwise epistasis among segments reflects their genetic incompatibilities. Here, epistasis imposes a fitness cost to reassortment, even between strains of the same subtype, and could limit host-range expansion [64,82,83]. 

### 3.3. Epistasis in the Adaptive Evolution of Influenza Virus

Most studies of epistasis in influenza virus have focused on its role in antigenic evolution. HA evolution is characterized by a series of mutations with little apparent change in antigenicity, forming an antigenic cluster, followed by a mutation that leads to significant antigenic drift, called a cluster transition. Models show that epistatic interactions among individually neutral mutations can explain this pattern of evolution [84,85,86]. The epistatic interactions in these antigenic clusters can lead to historical contingency. Mutations involved in a cluster transition also interact with mutations involved in the subsequent cluster transition, forming chains of interacting mutations [65]. These chains suggest that the fixation of each substitution is contingent on the fixation of prior substitutions. 

**Figure 3:**
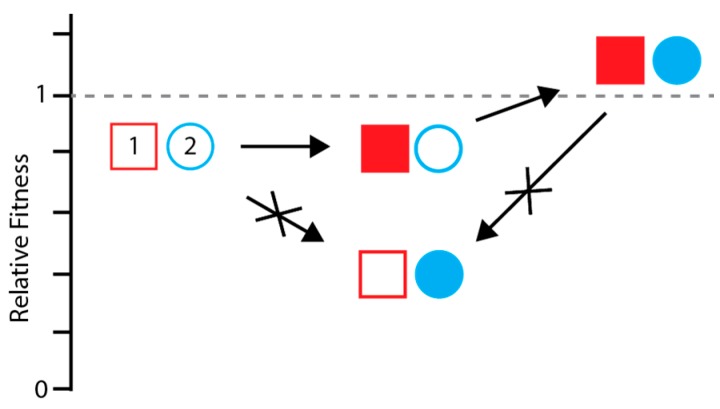
Epistasis can constrain adaptation. The ancestral identity of loci (1) and (2) are shown as unfilled red and blue shapes. A mutation at locus 2 mediates immune escape (filled circle) but is detrimental if it occurs on the ancestral background. Thus, a compensatory mutation at locus 1 (filled square) is required before the escape mutation, limiting the accessibility of the higher fitness genotype (filled square and filled circle). The compensatory mutation also becomes entrenched. Once the antigenic mutation arises, reversion of the compensatory mutation to its ancestral state (unfilled square) would cause a fitness decrease, even though it was initially neutral. Such interactions can occur within or between genes and involve more than two loci.

Studies employing site-directed mutagenesis and experimental evolution demonstrate how epistasis in HA leads to this historical contingency. First, the impact of a given mutation on antigenicity or receptor binding varies with genetic background [58,87]. This context dependence makes it harder to predict HA evolution and generalize molecular findings between strains. Second, mutations that mediate antigenic escape often have pleiotropic effects, and their success is contingent upon mutations that restore fitness. Antigenic escape variants in HA can decrease protein-folding stability or alter sialic acid binding [56,88,89,90,91,92], and fitness can be restored by mutations in HA or NA with opposing effects [56,61,89,90,92,93]. In many cases, the deleterious side effects of an antigenic mutation are larger than its beneficial effects. This constrains adaptation, as the novel, but deleterious, antigenic mutation can only be selected if a compensatory mutation arises first (Figure 3) [94]. Since many compensatory mutations are neutral, this often requires that the initial compensatory mutation arise by random drift, hitchhiking, or simultaneously with the novel antigenic mutation. 

The adaptive evolution of HA is also constrained by entrenchment, whereby a substitution can no longer revert to its ancestral state without compromising fitness (Figure 3). For example, Wu et al. found that a substitution in the receptor-binding site of H3, E190D, was reversible to the ancestral state within 10 years after the substitution arose, but not in more recent strains [57]. Apparently, more recent mutations in the receptor-binding site have altered its structure such that E190 is no longer tolerated. Interestingly, all of the epistatically interacting mutations were located in antigenic sites and could explain why mutations that lead to antigenic changes in HA rarely revert. 

Epistasis also influences the adaptive evolution of NA. NA phylogenies reveal chains of interacting substitutions similar to those in HA cluster transitions, and resistance to oseltamivir and other NA inhibitors is constrained by epistasis. Resistance mutations reduce fitness by altering NA stability or enzymatic activity and are contingent on compensatory mutations in HA or NA [53,62,93,95,96,97]. While oseltamivir was first introduced in 1999, the H274Y resistance mutation only arose eight years later in a much different genetic background [53,95,96]. It then swept the population in a single year. This single epistatic interaction demonstrates how the starting genotype of a strain can have profound effects on whether it can adapt to a new selective pressure.

There are fewer examples of epistasis in other influenza proteins. Contingency has been identified in M2 and NP phylogenies and immune escape mutations in NP [54,65]. We have found that mutations in PA and PB1 interact epistatically to mediate high-level resistance to mutagenic drugs in vitro [55]. Epistatic interactions in M2 may also mediate increased resistance to amantadine and/or increase virulence in amantadine-resistant strains [98,99,100]. For example, two mutations associated with amantadine resistance co-occur more frequently than predicted by chance [100]. This double mutant has become more prevalent in recent years [100] and has higher virulence in mice than either single mutant [99]. Finally, recent work suggests that selection on nonantigenic phenotypes encoded by the remaining six segments can have profound effects on antigenic evolution of the influenza virus [33,34]. Thus, defining epistasis across the genome is an important area for future study.

## 4. Conclusions

There are now extensive data on the effects of single mutations in influenza virus. The vast majority of mutations are deleterious, with similar effects as in other viruses. Greater mutational tolerance in some antigenic sites may enable their rapid evolution, whereas lower mutational tolerance makes other sites promising vaccine targets. The challenge now is to better understand the biological basis of mutational effects. Mutational fitness effects in the laboratory correlate with mutational frequency in nature [2,19]; thus, these data could be used to improve predictive models of influenza evolution [19,101,102]. 

In contrast, models and theoretical work on epistasis have outpaced empirical data. A handful of examples in several influenza genes demonstrate that epistasis is common and can lead to evolutionary flexibility via compensation, while at the same time constraining evolution through entrenchment and contingency. However, the general distribution of epistatic effects, including the sign and magnitude of epistasis, across the influenza genome is unknown. The general patterns of epistasis determine the likelihood of compensatory mutation and accessibility of adaptations, the consequences of reassortment, and thus the evolutionary fate of influenza populations. Novel methods are needed to investigate epistasis more extensively across the entire genome.
